# Proactive tobacco treatment offering free nicotine replacement therapy and telephone counselling for socioeconomically disadvantaged smokers: a randomised clinical trial

**DOI:** 10.1136/thoraxjnl-2015-207904

**Published:** 2016-03-01

**Authors:** Steven S Fu, Michelle van Ryn, David Nelson, Diana J Burgess, Janet L Thomas, Jessie Saul, Barbara Clothier, John A Nyman, Patrick Hammett, Anne M Joseph

**Affiliations:** 1 VA HSR&D Center for Chronic Disease Outcomes Research, Minneapolis, Minnesota, USA; 2 Department of Medicine, University of Minnesota Medical School, Minneapolis, Minnesota, USA; 3 Division of Health Care Policy & Research, Mayo Clinic, Rochester, Minnesota, USA; 4 North American Quitline Consortium, Phoenix, Arizona, USA; 5 Division of Health Policy and Management, University of Minnesota School of Public Health, Minneapolis, Minnesota, USA

**Keywords:** Smoking cessation, Tobacco control

## Abstract

**Background:**

Evidenced-based tobacco cessation treatments are underused, especially by socioeconomically disadvantaged smokers. This contributes to widening socioeconomic disparities in tobacco-related morbidity and mortality.

**Methods:**

The Offering Proactive Treatment Intervention trial tested the effects of a proactive outreach tobacco treatment intervention on population-level smoking abstinence and tobacco treatment use among a population-based sample of socioeconomically disadvantaged smokers. Current smokers (n=2406), regardless of interest in quitting, who were enrolled in the Minnesota Health Care Programs, the state's publicly funded healthcare programmes for low-income populations, were randomly assigned to proactive outreach or usual care. The intervention comprised proactive outreach (tailored mailings and telephone calls) and free cessation treatment (nicotine replacement therapy and intensive, telephone counselling). Usual care comprised access to a primary care physician, insurance coverage of Food and Drug Administration-approved smoking cessation medications, and the state's telephone quitline. The primary outcome was self-reported 6-month prolonged smoking abstinence at 1 year and was assessed by follow-up survey.

**Findings:**

The proactive intervention group had a higher prolonged abstinence rate at 1 year than usual care (16.5% vs 12.1%, OR 1.47, 95% CI 1.12 to 1.93). The effect of the proactive intervention on prolonged abstinence persisted in selection models accounting for non-response. In analysis of secondary outcomes, use of evidence-based tobacco cessation treatments were significantly greater among proactive outreach participants compared with usual care, particularly combination counselling and medications (17.4% vs 3.6%, OR 5.69, 95% CI 3.85 to 8.40).

**Interpretation:**

Population-based proactive tobacco treatment increases engagement in evidence-based treatment and is effective in long-term smoking cessation among socioeconomically disadvantaged smokers. Findings suggest that dissemination of population-based proactive treatment approaches is an effective strategy to reduce the prevalence of smoking and socioeconomic disparities in tobacco use.

**Trial registration number:**

NCT01123967.

**Key messages**

**What is the key question?**

- What is the effect of population-based proactive tobacco treatment on use of evidence-based smoking cessation treatments and long-term quit rates compared with usual care among socioeconomically disadvantaged smokers?

**What is the bottom line?**

- Population-based proactive tobacco treatment is effective in increasing engagement in evidence-based tobacco cessation treatments and for increasing long-term population quit rates among hard to reach, socioeconomically disadvantaged smokers.

**Why read on?**

- Taken together with prior research, these findings suggest that the dissemination and large-scale adoption of proactive tobacco treatment approaches may reduce smoking prevalence and socioeconomic disparities in tobacco use.

## Background

Smoking rates are much higher in socioeconomically disadvantaged populations in the majority of developed countries.^1^ Among those experiencing multiple forms of disadvantage (eg, single-parent households, public housing, no access to a car, etc), smoking rates can be as high as 60%, while rates among the most affluent can be as low as 15%.^2^ ^3^ In the USA, 28% of adults living below the federal poverty line smoke cigarettes compared with 17% of adults at or above the poverty level.^4^ Among adults younger than 65, 16% of those with private health insurance are current smokers, compared with 34% of Medicaid (a government public health insurance programme) recipients and 32% of the uninsured.^5^ Because socioeconomically disadvantaged populations smoke more than their more advantaged counterparts, they also suffer disproportionately from smoking-caused diseases.

With respect to smoking cessation, socioeconomically disadvantaged smokers are less likely to use evidence-based smoking cessation treatments (pharmacotherapy including nicotine replacement therapy (NRT), bupropion and varenicline and/or counselling either in-person or by telephone) than the general population of smokers.^6–9^ Contributing factors to the disparity in evidence-based treatment use include greater life stressors that reduce motivation to quit, lack of knowledge about the benefits of using pharmacotherapy and lack of awareness regarding Medicaid coverage for smoking cessation treatment.^10^ ^11^ Other barriers include a lower likelihood of receiving preventive care services, difficulty taking time from work for cessation services, travel time and costs and an inability to pay out-of-pocket expenses for pharmacotherapy.^12^ Providers are also less likely to offer smoking cessation treatment to low-income smokers, perhaps related to provider bias or negative assumptions about interest in quitting.^13^ ^14^

The objective of this randomised controlled trial was to test whether a proactive tobacco treatment intervention, designed to overcome these critical barriers to access and delivery of evidence-based smoking cessation treatment, would improve smoking cessation outcomes relative to usual care among a population-based sample of publicly insured smokers. We chose a population-based approach to fully examine effects among all smokers in the cohort, not just those who expressed interest in quitting.

## Methods

### Study design and participants

The Offering Proactive Treatment Intervention (OPT-IN) study was approved by the Institutional Review Boards at the University of Minnesota and the Minnesota Department of Human Services (DHS). As previously described,^15^ the study was a two-group randomised controlled trial conducted among clients of the Minnesota Health Care Programs (MHCP) (Medicaid or MinnesotaCare). Medicaid is a joint federal-state programme that provides payment for medical care for people falling into certain categories, including poverty and certain disabilities. MinnesotaCare is for Minnesota residents without access to affordable healthcare coverage, but who have higher income than those covered by Medicaid/Medical Assistance. The sampling frame consisted of a random sample of non-institutionalised MHCP clients that was stratified by age group (18–24, 25–34, and 35–64), by gender and by MHCP. If two or more individuals had the exact same address, one individual was randomly selected to be in the sample. Study participants were recruited using a mailed, baseline tobacco use screening survey packet that included an informed consent statement and notice of privacy practices. Participants were informed that if they were currently smoking and returned a completed baseline survey they would be participating in a research study and would have a 50% chance of being offered a new smoking cessation programme.

### Randomisation and masking

Individuals who returned a completed baseline survey and reported current cigarette smoking (defined as having smoked a cigarette in the past 30 days, even a puff) were randomised, with equal likelihood within each of the 12 age, gender and MHCP strata (Medicaid or MinnesotaCare), to receive either (1) proactive outreach intervention or (2) usual care. The target recruitment goal was 2500 current cigarette smokers. In contrast to aid-to-cessation trials testing the efficacy of an intervention in smokers interested in quitting, this population impact trial of smoking cessation outreach and treatment included all identified smokers, regardless of their interest in quitting.^16–18^ Participants were not blinded. However, study staff who administered the questionnaires to collect primary outcome data were blinded to participant's treatment allocation.

### Procedures

#### Usual care

All MHCP enrolees are assigned a primary care provider and usual care participants could contact their provider to access smoking cessation treatment. However, tobacco treatment was variable and depended on the primary care provider's willingness and capacity to adhere to guidelines. Usual care participants also had access to smoking cessation medications (NRT, sustain-released bupropion or varenicline) at substantially reduced cost ($1–$5 co-pay) through MHCP insurance coverage by obtaining a prescription from their provider. Alternatively, participants could purchase over-the-counter NRT at retail costs. In addition, they could access free telephone counselling by calling the Minnesota state quitline (1-888-354-PLAN).

#### Proactive outreach intervention

Intervention participants were able to receive the same MHCP provider smoking cessation treatment components as usual care participants. Additionally, the proactive outreach tobacco treatment intervention included two primary elements: (1) personalised mailings and telephone calls and (2) facilitated access to a free, comprehensive, evidence-based treatment for tobacco dependence (NRT and intensive, telephone-based behavioural counselling). We designed the intervention to overcome both access barriers and psychosocial barriers experienced by socioeconomically disadvantaged smokers, which was delivered by study telephone counsellors trained in motivational interviewing and smoking cessation counselling.

Personalised mailings included invitation materials: a letter and brochure describing the University of Minnesota Choose to Quit Smoking cessation programme and the services available to help MHCP enrolees quit smoking. Approximately 3 weeks later, study counsellors called participants with up to 12 contact attempts made at different times of the day over 4 weeks. The choice of 12 contact attempts was based on experience of our pilot of intervention, which was an increase from six call attempts in the original protocol. The purpose of the outreach call was to (1) deliver motivational advice to quit smoking, (2) promote self-efficacy, (3) encourage participants to engage in smoking cessation treatment and (4) provide information on the safety, efficacy and functional benefits of pharmacotherapy, particularly NRT. Employing motivational interviewing techniques, counsellors tailored the content of the call to the participant's readiness to quit and concerns about quitting.^19^ Motivational interviewing is patient-centred and an evidence-based counselling practice directed at identifying ambivalence and enhancing intrinsic motivation for behavioural change.^20^ ^21^

After the outreach call, telephone care comprised free proactive telephone counselling, free NRT and a self-help quit smoking manual. Specifically, study counsellors used an adaptation of the evidence-based California Helpline protocol which consisted of seven calls initiated by the counsellor, scheduled in a relapse-sensitive fashion over a 2-month period for those ready to set a quit date (pre-quit, quit day, then 3 days, 1 week, 2 weeks, 1 months and 2 months after the quit date).^22^ Given variability in participants’ readiness to quit and prior experience with quitting, counselling calls were individually tailored to address the participant's needs. For example, participants who were thinking about quitting but not ready to set a quit date right away received motivational interviewing to enhance their readiness to quit and the call schedule was based on participant preferences. In addition, participants who relapsed to smoking were encouraged to set new quit dates and repeat the counselling programme. In total, a participant was eligible to receive up to 14 counselling calls.

Participants were also provided a free 8-week course of NRT (patch, gum or lozenge). NRT was mailed directly to participants in anticipation of their quit date using a protocol based on the US Public Health Service Guideline recommendations.^9^ ^23^ NRT was purchased from the GlaxoSmithKline Consumer Health Care Government Customer Direct Purchase Program. All participants who received telephone counselling were offered NRT unless they had one of the following contraindications: (1) recent (within 2 weeks) heart attack or severe arrhythmia, (2) unstable angina or (3) pregnancy. Participants were not required to participate in telephone counselling in order to receive NRT, although this practice was not promoted. Participants interested in bupropion or varenicline were referred to their primary care provider. Participants who relapsed and attempted to quit again were able to receive an additional 4 weeks of NRT.

#### Data collection

There were two episodes of data collection: baseline and 1 year following randomisation (participant surveys and DHS administrative data). The baseline and follow-up surveys used modified Dillman mail survey procedures and have been previously described.^15^ The 1-year follow-up survey followed similar procedures as the mailed baseline survey and included additional procedures to reduce attrition. These included a $10.00 incentive with the first mailing, telephone administration (mixed-mode protocol) for non-respondents to the mailed protocol, and tracking procedures for non-respondents including receipt of updated contact information from the Minnesota DHS.

### Outcomes

The primary outcome was self-reported 6-month prolonged smoking abstinence at 1 year following randomisation. A person who smoked at least once on seven consecutive days or who smoked at least once on two consecutive weekends in the 6-month period was defined as a treatment failure. The choice and definition of the primary outcome follows recommendations of the Society for Research on Nicotine and Tobacco Measures Workgroup to report multiple measures of abstinence in which prolonged abstinence is the preferred measure.^18^ Since OPT-IN was a cessation-induction trial (ie, evaluation of an intervention to encourage cessation among a population-based sample of smokers, including those not currently trying to quit), follow-up was tied to the onset of the intervention (ie, time of randomisation). Secondary outcomes included self-reported 30-day point prevalence abstinence and 7-day point prevalence abstinence, use of behavioural counselling, use of smoking cessation medications and use of combination counselling and medication.

### Statistical analysis

As previously described,^15^ the goal sample size for this study was 2500 participants (1250 per group), which accounted for attrition in order to have observed smoking abstinence outcomes on 1500 respondents (750 per group). This sample size provides approximately 85% power or greater with a two-sided α of 0.05 to detect differences if the intervention raises quit rates by 4%.

Baseline data were obtained for all participants (n=2406) using a baseline survey and DHS administrative records. The usual care (n=1206) and proactive outreach (n=1200) groups were compared across the stratification variables of age, sex and insurance type, as well as socio-demographic and smoking-related clinical variables presented in table 1 using Pearson's χ^2^ tests and two-sample t tests. Logistic regressions, adjusted for the stratification variables of age, sex and insurance type, modelled the odds of treatment use over the year of follow-up (table 2). Separate regression models were used for each treatment use outcome within the medication, counselling and combination categories.

**Table 1:** Baseline demographic and smoking characteristics, according to treatment group

Characteristic	Usual care (n=1206)	Proactive outreach (n=1200)	Total (n=2406)
*Demographics*			
Insurance programme			
Medicaid	878 (72.8%)	871 (72.6%)	1749 (72.7%)
MinnesotaCare	328 (27.2%)	329 (27.4%)	657 (27.3%)
Gender (female)	853 (70.7%)	846 (70.5%)	1699 (70.6%)
Age category			
18–24	249 (20.7%)	247 (20.6%)	496 (20.6%)
25–34	414 (34.3%)	410 (34.2%)	824 (34.3%)
35–64	543 (45.0%)	543 (45.3%)	1086 (45.1%)
Race/ethnicity			
Non-Hispanic white	944 (78.3%)	941 (78.4%)	1885 (78.4%)
Black	122 (10.1%)	134 (11.2%)	256 (10.6%)
American Indian	87 (7.2%)	80 (6.7%)	167 (6.9%)
Hispanic	19 (1.6%)	23 (1.9%)	42 (1.8%)
Asian	34 (2.8%)	22 (1.8%)	56 (2.3%)
Education			
Grade 11/lower	156 (13.2%)	166 (14.1%)	322 (13.7%)
HS grad/GED	383 (32.5%)	398 (33.9%)	781 (33.2%)
Some college	487 (41.3%)	490 (41.7%)	977 (41.5%)
College grad/higher	154 (13.1%)	120 (10.2%)	274 (11.6%)
Employment			
Employed/self-employed	608 (51.2%)	598 (51.0%)	1206 (51.1%)
Student	75 (6.3%)	87 (7.4%)	162 (6.9%)
Out of work	154 (13.0%)	153 (13.0%)	307 (13.0%)
Unable to work/disabled	276 (23.2%)	277 (23.6%)	553 (23.4%)
Homemaker	75 (6.3%)	58 (4.9%)	133 (5.6%)
Yearly income			
Less than $10k	427 (36.9%)	430 (37.7%)	857 (37.3%)
$10 001 to $20k	345 (29.8%)	375 (32.9%)	720 (31.4%)
$20 001 to $40k	259 (22.4%)	233 (20.4%)	492 (21.4%)
More than $40k	125 (10.8%)	103 (9.0%)	228 (9.9%)
Child in home	665 (56.2%)	651 (55.6%)	1316 (55.9%)
Smoking			
Cigarettes/day	13.8 (9.1)	13.4 (9.2)	13.6 (9.2)
Time until first cigarette (min)			
≤5	321 (26.6%)	296 (24.7%)	617 (25.6%)
6–30	536 (44.4%)	538 (44.8%)	1074 (44.6%)
≥30	349 (28.9%)	366 (30.5%)	715 (29.7%)
Cigarette type			
Menthol	450 (37.5%)	442 (37.1%)	892 (37.3%)
Non-menthol	750 (62.5%)	751 (63.0%)	1501 (62.7%)
Motivation to quit	6.3 (2.8)	6.3 (2.9)	6.3 (2.9)
Quit attempt (past year)	643 (54.0%)	644 (54.6%)	1287 (54.3%)
Treatment used (past year)			
Counselling only	10 (0.8%)	9 (0.8%)	19 (0.8%)
Medication only	325 (27.0%)	337 (28.1%)	662 (27.5%)
Both	33 (2.7%)	38 (3.2%)	71 (3.0%)
Neither	838 (69.5%)	816 (68.0%)	1654 (68.7%)

Data are number (%) or mean (SD). Motivation to quit assessed using the contemplation ladder which asked participants to indicate their readiness to quit on a scale from 0 to 10, with higher values indicative of greater readiness to quit.^24^ A value of 0 corresponds with the statement, ‘No thought of quitting’, a value of 5 corresponds with the statement, ‘Think I should quit but not quite ready’ and a value of 10 corresponds with the statement, ‘Taking action to quit’ (eg, cutting down, enrolling in a programme). GED, General Educational Development; grad, graduation; HS, High school.

**Table 2:** Treatment usage by treatment group over the 1-year follow-up period

Treatment type	Usual care (n=944)	Proactive outreach (n=826)	OR*	p Value
Medication				
Any medication†	278 (29.5%)	335 (40.6%)	1.63 (1.34–2.00)	<0.001
NRT	192 (20.5%)	275 (33.8%)	1.99 (1.60–2.48)	<0.001
Bupropion/varenicline	104 (11.1%)	105 (12.9%)	1.19 (0.89–1.59)	0.249
Counselling				
Any counselling	45 (4.8%)	174 (21.1%)	5.42 (3.83–7.66)	<0.001
Phone	27 (2.9%)	155 (19.4%)	8.08 (5.29–12.33)	<0.001
In-person	23 (2.6%)	72 (9.4%)	3.87 (2.38–6.29)	<0.001
Combination				
None reported	655 (69.4%)	461 (55.8%)	0.55 (0.45–0.68)	<0.001
Medication only	244 (25.9%)	191 (23.1%)	0.85 (0.68–1.06)	0.150
Counselling only	11 (1.2%)	30 (3.6%)	3.21 (1.60–6.47)	0.001
Medication and counselling	34 (3.6%)	144 (17.4%)	5.69 (3.85–8.40)	<0.001
Any cessation treatment used	289 (30.6%)	365 (44.2%)	1.81 (1.48–2.21)	<0.001

Data are n (%) or OR (95% CI).

*Adjusted for stratification variables of age, sex and insurance type.

†Participants could report using more than one medication.

NRT, nicotine replacement therapy.

For the analysis of our primary outcome, we fit a stratified logistic regression equation modelling the odds that a participant reported 6-months prolonged abstinence at 1-year follow-up using intervention group, age, gender and MHCP strata as explanatory variables (table 3). Similar analyses assessed the effect of the proactive intervention on reported 30-day and 7-day abstinence. The initial analyses used data from those who responded to the follow-up survey. To address potential informative non-response bias in these initial analyses, we fit a series of selection model analyses. Two common, related, approaches for addressing informative missing data comprise selection models and pattern mixture models.^25^ Selection models jointly model the study outcomes and the missing of the outcomes, for all participants, by modelling (1) how outcomes are related to the available predictors and (2) modelling how whether the outcome measure is missing is related to the value of the outcome measure and the available predictors. We posited different assumptions for how follow-up survey response would be related to abstinence, the sampling strata and selected covariates. A given selection model analysis jointly fit the two models:

**Table 3:** Smoking abstinence by treatment group at 1 year

	Model-based estimate of association			
Abstinence outcome	Usual care abstinence rate	Proactive outreach abstinence rate	OR (95% CI)	p Value
6 month prolonged				
Analysis of observed data*	12.1% (113/937)	16.5% (135/820)	1.47 (1.12 to 1.93)	0.006
Selection model analysis†	7.8–9.0%	11.2–14.2%	1.50 to 1.68	<0.001–0.002
30-day point prevalence				
Analysis of observed data*	12.1 (114/940)	15.0 (124/826)	1.31 (0.99 to 1.73)	0.055
Selection model analysis†	7.7–7.8%	10.1–10.1%	1.33 to 1.34	0.030–0.033
7-day point prevalence				
Analysis of observed data*	16.3% (154/942)	17.4% (143/823)	1.11 (0.86 to 1.42)	0.439
Selection model analysis†	11.1–11.6%	11.2–11.5%	0.99 to 1.05	0.719–0.932

*Data are percentages (n/N) with model estimated OR (95% CI) for the intervention and corresponding p value from logistic regression of abstinence on intervention adjusted for age, sex and insurance type stratification measures.

†Results presented are the range of least square mean type estimated percentages with range of estimate ORs for intervention and corresponding p values from the regression models for the abstinence outcome with lower AIC statistics.


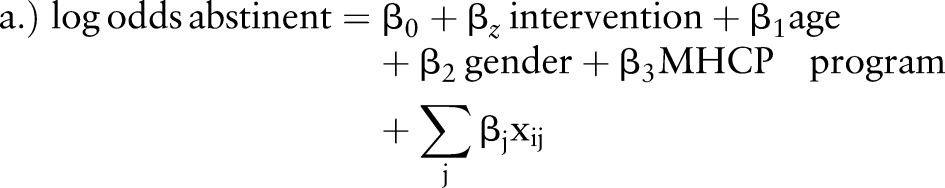


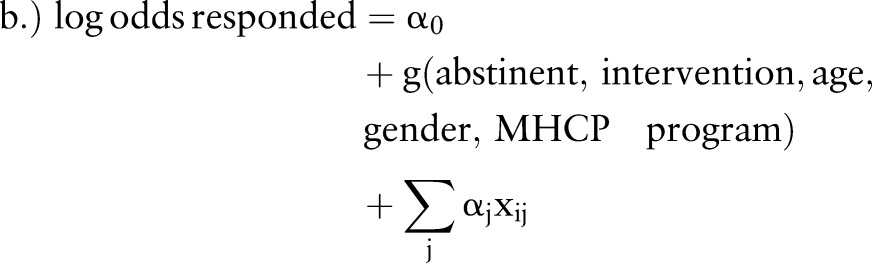

for a specified function *g* and selected set of covariates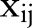 for the *i*th participant, to the observed data for all participants using the expectation–maximisation algorithm process proposed by Ibrahim and Lipsitz.^25^ For the specification of the function *g*, the series of selection models considered (1) a simple additive model, (2) a model adding an interaction between intervention and abstinence to this simple additive model and (3) seven different models adding different combinations of interactions between abstinence and the sampling strata to the model with the interaction between abstinence and intervention. In addition to age, gender and healthcare coverage programme strata, the models incorporated patient demographics, smoking history, quit attempt history and motivation to quit and measures of general health, alcohol use and mental health including those measures that differed between respondents and non-respondents to the follow-up survey. (Additional details can be found in the online supplementary appendix.)

10.1136/thoraxjnl-2015-207904.supp1 Supplementary data

### Role of the funding source

The funder of the study had no role in study design, data collection, data analysis, data interpretation or writing of the report. The corresponding author had full access to all the data in the study and had final responsibility for the decision to submit for publication.

## Results

### Study participants

**Figure 1:**
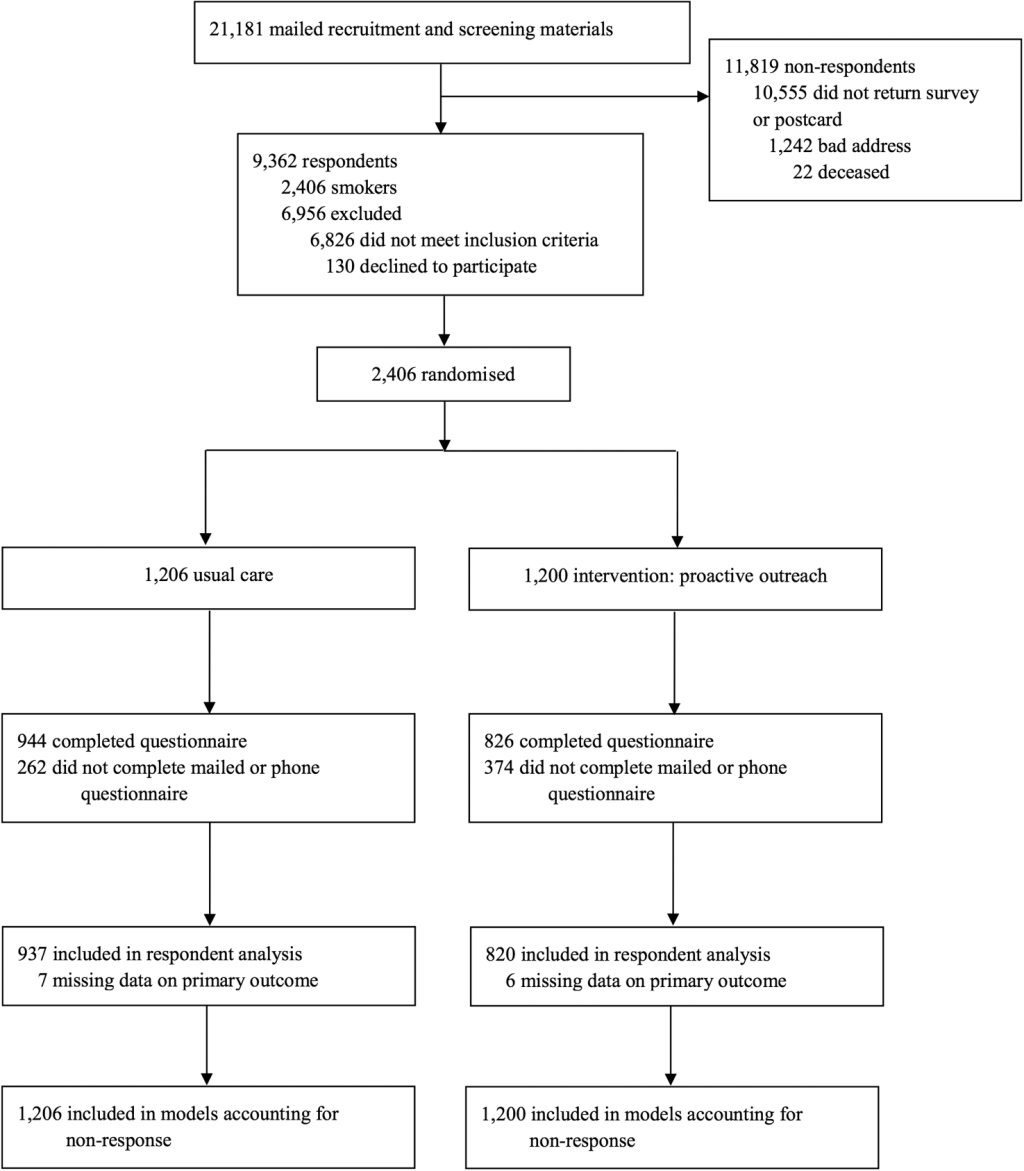
Flow chart showing the enrolment, randomisation and follow-up of the study participants.

Study participants were recruited from July 2011 to August 2012. We mailed recruitment and tobacco use screening surveys to 21 181 MHCP clients. There were 9362 respondents of whom 2406 were current smokers (25.7%) (see figure 1). The blocked randomisation, implemented separately within the 12 age, gender and MHCP strata, assigned 1200 participants to proactive care and 1206 participants to usual care. The overall follow-up survey response rate was 74% (69% proactive outreach vs 78% usual care). As illustrated in figure 1, complete primary outcome data were available for 820 participants in proactive outreach (68%) and 937 participants in usual care (78%) and were used for the respondent analysis. All randomised participants (n=2406) were included in the selection models accounting for non-response. There were no significant differences in baseline characteristics between the intervention and usual care arms (table 1).

### Proactive outreach engagement

Telephone outreach was successful in contacting 836 participants (70%) in the proactive treatment group. Among those who participated in the outreach call, 49 (6%) had already quit smoking, 397 (47%) expressed interest in participating in telephone coaching and 291 (34%) subsequently completed a counselling call. Among this latter group, 212 (73%) were ready to set a quit date, 79 (27%) wanted to talk more about their smoking before setting a quit date and the average number of total completed telephone counselling calls per participant was 4.7.

### Tobacco treatment use

Table 2 details tobacco treatment use by the intervention and usual care arms. Proactive outreach participants were much more likely to use smoking cessation medications compared with usual care (40.6% vs 29.4%), particularly NRT. Telephone counselling for smoking cessation was higher in the proactive outreach intervention group compared with usual care (19.4% vs 2.9%). Furthermore, the rate of combined behavioural counselling and medication treatment was significantly higher in the proactive outreach intervention group (17.4% vs 3.6%, OR 5.69 (95% CI 3.85 to 8.40)).

### Smoking abstinence

Participants in the proactive outreach intervention group were significantly more likely to quit smoking than usual care participants. The primary outcome, 6-month prolonged smoking abstinence rate at 1 year, was significantly higher for proactive outreach compared with usual care (16.5% vs 12.1%, OR 1.47 (95% CI 1.12 to 1.93)) (table 3). The number needed to treat (NNT) to gain one participant with 6-month prolonged abstinence was 23. In table 3, we present the selection model analyses which consist of a range of estimated ORs for intervention and estimated abstinence rates across the series of fitted models. The selection models with lower Akaike information criterion (AIC) statistics for modelling smoking abstinence yielded the more plausible estimated abstinence rates. Among these models, estimated prolonged abstinence rates were lower than in the observed data but the differences in rates between outreach and usual care were generally consistent with the observed difference with a range of 3.3%–5.2% while the estimated ORs ranged from 1.50 (1.15 to 1.96) to 1.68 (1.319 to 2.16). Among secondary outcomes, similar effects were found for 30-day abstinence favouring the proactive outreach intervention in analysis of observed data and selection models. However, results for 7-day point prevalence abstinence were insignificant.

## Discussion

Proactive outreach was effective at markedly increasing the use of tobacco cessation treatments among socioeconomically disadvantaged smokers, particularly telephone counselling and the combination of counselling and medication. Furthermore, the proactive tobacco treatment intervention was effective at increasing long-term quit rates compared with usual care. This randomised controlled trial adds evidence supporting the effectiveness of proactive tobacco treatment for increasing the population impact of tobacco cessation treatment. An absolute increase in long-term smoking cessation rates of 4.4% is highly significant from a public health perspective. In the USA, the Medicaid programme provides health coverage for 11 million non-elderly low-income adults^27^ and assuming a 34% prevalence of current smoking an estimated population of 3.74 million smokers.^5^ ^28^ If such results were generalised to the entire current smoking Medicaid population, there would be nearly 165 000 fewer cigarette smokers in Medicaid.

The current study contributes new evidence to support the use of population-based proactive approach to deliver tobacco cessation treatment and confirms that this approach is feasible, effective and applicable to diverse settings and populations. Rigotti *et al* found increased NRT use (OR 3.47) and short-term abstinence (7-day point prevalence abstinence at 3 months, 5.3% vs 1.1%, OR 5.35) using free telephone consultation with a tobacco coordinator providing 8 weeks of NRT and proactive referral to a state quitline.^29^ Fu *et al* found higher 6-month prolonged abstinence at 1 year (13.5% vs 10.9%, OR 1.27) compared with usual care among veteran smokers using proactive outreach programme offering telephone coaching and Veterans Health Administration smoking cessation services.^30^ Using interactive voice response technology to proactively offer telephone counselling, free NRT and community-based referrals among a disadvantaged population, Haas *et al* found increased 7-day abstinence at 9 months (17.8% vs 8.7%, OR 2.5).^31^

Those engaging in telephone counselling completed an average 4.7 counselling calls, which is substantially higher than the average number of calls reported by publicly funded quitlines.^32^ This is striking given that for most state-based quitlines, initial contact to the quitline requires action on the part of the tobacco user indicating a relatively high degree of interest in using the quitline's services. This study recruited tobacco users at all stages of readiness to quit. It may be that there was previously unmet demand for such services that the proactive outreach approach made more accessible or salient. It may also be that tobacco users who were not yet ready to make a quit attempt at the start of the study responded well to opportunities to discuss the quitting process and were willing to engage in a higher number of counselling interactions by phone. The increase in use of quitting medications is understandable given the perceived lack of access to free or low-cost medication alternatives for the study population. Despite state-wide media campaigns promoting the Minnesota QUITPLAN Helpline during the study period, it may be that the proactive approach used for the study was particularly effective at increasing awareness of, and interest in, using evidence-based cessation tools.

We observed significant intervention effects on 6-month prolonged abstinence, the primary outcome, and 30-day abstinence, a secondary outcome. However, we did not observe significant effects on 7-day point prevalence abstinence, which was another secondary outcome. Since 7-day point prevalence abstinence also includes participants who made a quit attempt after the intervention period, this measure may have underestimated the effectiveness of the proactive outreach intervention. Alternatively, this may suggest that usual care participants were able to achieve similar short-term abstinence but not sustained abstinence, perhaps related to the lower use of treatment by the usual care group.

This study has several limitations. First, smoking abstinence outcome relied on self-report and was not biochemically verified; however, this approach is similar to other population-based interventions.^33^ In addition, biochemical verification is not possible for 6-month prolonged abstinence, the study's primary outcome. Second, the follow-up survey response rate was 74%. While this is an excellent response rate considering the low socioeconomic characteristics of the population, there was differential response by intervention and usual care arms and potential for non-response bias. We conducted a series of selection model analyses to account for non-response and observed similar effects, suggesting that our findings are robust. Third, the intervention consisted of a discrete episode of care and it is possible a longitudinal or chronic disease model of care for tobacco use would be more effective. Further research is needed to assess the effects of proactive treatment as part of chronic disease management. Fourth, it is possible that there could have been a Hawthorne effect for usual care participants due to their awareness of and participation in the study as a result of completing the baseline survey. In the unlikely event that the baseline survey did exert a therapeutic effect, it would have been conservative and attenuated the observed effects. Finally, our study did not include high socioeconomic groups and it is unknown if the intervention would have differential effects between income population groups. According to the ‘fundamental cause’ perspective on health disparities, public health interventions that reduce morbidity and mortality can create health disparities because advantaged groups are often better poised to take advantage of opportunities afforded by the intervention. In light of evidence supporting the fundamental cause perspective, it might be advisable to target interventions such as the current proactive outreach intervention to socioeconomically disadvantaged groups to reduce tobacco-related health disparities.

In conclusion, this population-based clinical trial demonstrates the effectiveness of proactive tobacco treatment for increasing engagement in evidence-based tobacco cessation treatments and for increasing long-term population quit rates among hard to reach, socioeconomically disadvantaged smokers. Results of this trial may be of particular interest for public insurance agencies, European quitlines and the North American Quitline Consortium, a membership organisation for all publicly funded quitlines in the USA and Canada, whose goals are to maximise the effectiveness, broaden the reach and increase service capacity of quitlines. Administrators of publicly funded quitlines, public insurance agencies (eg, Medicaid) and other health-related organisations should consider adopting a proactive outreach strategy for tobacco users in addition to mass media promotions as a way of effectively recruiting and enrolling socioeconomically disadvantaged tobacco users to smoking cessation services.

## References

[1] Hiscock R, Bauld L, Amos A, et al Socioeconomic status and smoking: a review. Ann N Y Acad Sci 2012;1248:107–23. 10.1111/j.1749-6632.2011.06202.x 22092035

[2] Amos A, Bauld L, Clifford D, et al Tobacco control, inequalities in health and action at a local level. York: Public Health Research Consortium, 2011.

[3] Sharma A, Lewis S, Szatkowski L Insights into social disparities in smoking prevalence using Mosaic, a novel measure of socioeconomic status: an analysis using a large primary care dataset. BMC Public Health 2010;10:755 10.1186/1471-2458-10-755 21138555 PMC3016386

[4] Ahmed J, Agaku IT, O'Connor E, et al Current cigarette smoking among adults—United States, 2005–2013. MMWR Morb Mortal Wkly Rep 2014;63:1108–12. 25426653 PMC5779518

[5] Schiller JS, Lucas JW, Ward BW, et al Summary health statistics for U.S. adults: National Health Interview Survey, 2010. Vital Health Stat 10 2012;252:1–207. 22834228

[6] Fu SS, Sherman SE, Yano EM, et al Ethnic disparities in the use of nicotine replacement therapy for smoking cessation in an equal access health care system. Am J Health Promot 2005;20:108–16. 10.4278/0890-1171-20.2.108 16295702

[7] Fu SS, Kodl MM, Joseph AM, et al Racial/Ethnic disparities in the use of nicotine replacement therapy and quit ratios in lifetime smokers ages 25 to 44 years. Cancer Epidemiol Biomarkers Prev 2008;17:1640–7. 10.1158/1055-9965.EPI-07-2726 18583471 PMC2593846

[8] Cokkinides VE, Ward E, Jemal A, et al Under-use of smoking-cessation treatments: results from the National Health Interview Survey, 2000. Am J Prev Med 2005;28:119–22. 10.1016/j.amepre.2004.09.007 15626567

[9] Fiore MC, Jaen CR, Baker TB, et al Treating tobacco use and dependence: 2008 update. Clinical Practice Guideline. Rockville, MD: USDHHS, Public Health Service, 2008.

[10] McMenamin SB, Halpin HA, Ibrahim JK, et al Physician and enrollee knowledge of Medicaid coverage for tobacco dependence treatments. Am J Prev Med 2004;26:99–104. 10.1016/j.amepre.2003.10.017 14751319

[11] McMenamin SB, Halpin HA, Bellows NM Knowledge of Medicaid coverage and effectiveness of smoking treatments. Am J Prev Med 2006;31:369–74. 10.1016/j.amepre.2006.07.015 17046407

[12] Blumenthal DS Barriers to the provision of smoking cessation services reported by clinicians in underserved communities. J Am Board Fam Med 2007;20:272–9. 10.3122/jabfm.2007.03.060115 17478660

[13] Browning K, Ferketich A, Salsberry P, et al Socioeconomic disparity in provider-delivered assistance to quit smoking. Nicotine Tob Res 2008;10:55–61. 10.1080/14622200701704905 18188745

[14] Houston T, Scarinci I, Person S, et al Patient smoking cessation advice by health care providers: the role of ethnicity, socioeconomic status, and health. Am J Public Health 2005;95:1056–61. 10.2105/AJPH.2004.039909 15914833 PMC1449308

[15] Fu SS, Van ryn M, Burgess DJ, et al Proactive tobacco treatment for low income smokers: study protocol of a randomized controlled trial. BMC Public Health 2014;14:337 10.1186/1471-2458-14-337 24716466 PMC3995758

[16] Abrams DB, Orleans CT, Niaura RS, et al Integrating individual and public health perspectives for tobacco treatment under managed health care: a combined stepped-care and matching model. Ann Behav Med 1996;18:290–304. 10.1007/BF02895291 18425675

[17] Glasgow RE, Vogt TM, Boles SM Evaluating the public health impact of health promotion interventions: the RE-AIM framework. Am J Public Health 1999;89:1322–7. 10.2105/AJPH.89.9.1322 10474547 PMC1508772

[18] Hughes JR, Keely JP, Niaura RS, et al Measures of abstinence in clinical trials: issues and recommendations. Nicotine Tob Res 2003;5:13–25. 10.1080/1462220031000070552 12745503

[19] DiClemente CC Motivational interviewing and the stages of change. In: Miller WR, Rollnick S Motivational interviewing: preparing people to change addictive behavior. New York: Guilford Press, 1991:191–202.

[20] Rollnick S, Miller W, Butler C Motivational interviewing in health care. New York, NY: Guilford Press, 2008.

[21] Lai DT, Cahill K, Qin Y, et al Motivational interviewing for smoking cessation. Cochrane Database Syst Rev 2010;(1):CD006936. 20091612 10.1002/14651858.CD006936.pub2

[22] Zhu SH, Anderson CM, Tedeschi GJ, et al Evidence of real-world effectiveness of a telephone quitline for smokers: comment. NEJM 2002;347:1087–93. 10.1056/NEJMsa020660 12362011

[23] Fiore MC, Jaen CR A clinical blueprint to accelerate the elimination of tobacco use. JAMA 2008;299:2083–5. 10.1001/jama.299.17.2083 18460668

[24] Biener L, Abrams DB The contemplation ladder: validation of a measure of readiness to consider smoking cessation. Health Psychol 1991;10:360–5. 10.1037/0278-6133.10.5.360 1935872

[25] Ibrahim JG, Lipsitz SR Parameter estimation from incomplete data in binomial regression when the missing data mechanism is nonignorable. Biometrics 1996;52:1071–8. 10.2307/2533068 8805768

[26] Little RJA Selection and pattern mixture models. In: Fitzmaurice G, Davidian M, Verbeke G, et al eds. Advances in longitudinal data analysis. London, UK: CRC Press, 2008:409–31.

[27] Non-Disabled Adults. Medicaid.gov. https://www.medicaid.gov/medicaid-chip-program-information/by-population/adults/non-disabled-adults.html (accessed 23 Dec 2015).

[28] Tobacco Cessation. Medicaid.gov. https://www.medicaid.gov/Medicaid-CHIP-Program-Information/By-Topics/Benefits/Tobacco.html (accessed 23 Dec 2015).

[29] Rigotti NA, Bitton A, Kelley JK, et al Offering population-based tobacco treatment in a healthcare setting: a randomized controlled trial. Am J Prev Med 2011;41:498–503. 10.1016/j.amepre.2011.07.022 22011421 PMC3235408

[30] Fu SS, Van ryn M, Sherman SE, et al Proactive tobacco treatment and population-level cessation: a pragmatic randomized clinical trial. JAMA Intern Med 2014;174:671–7. 10.1001/jamainternmed.2014.177 24615217

[31] Haas JS, Linder JA, Park ER, et al Proactive tobacco cessation outreach to smokers of low socioeconomic status: a randomized clinical trial. JAMA Intern Med 2015;175:218–26. 10.1001/jamainternmed.2014.6674 25506771 PMC4590783

[32] 2013. http://www.naquitline.org/?page=2012Survey (accessed 5 May 2015).

[33] SRNT Subcommittee on Biochemical Verification. Biochemical verification of tobacco use and cessation. Nicotine Tob Res 2002;4:149–59. 12028847 10.1080/14622200210123581

