# Supplementary material for: Proactive tobacco treatment offering free nicotine replacement therapy and telephone counselling for socioeconomically disadvantaged smokers: a randomised clinical trial
Source: Thorax. 2016 Mar 1;71(5):446–53. doi: 10.1136/thoraxjnl-2015-207904 (PMC4862067; doi:10.1136/thoraxjnl-2015-207904)
Supplement: Supplementary data [file thoraxjnl-2015-207904supp.pdf]

Details concerning Selection Model Analyses

We had originally planned to do both an imputation based assessment and a pattern-mixture/selection model based assessment of the potential presence and impact of response bias but instead implemented only a selection based approach. The planned imputation methods would have modeled survey response as a function of baseline characteristics and used the developed model to adjust the analysis for identified differences in response. As the selection models we employed also modeled differences in response between the groups using these baseline characteristics together with potential abstinence outcomes and these models were more general, we decided to omit the imputation based approach.

Background on Selection Model Analyses. In the study considered here we have binary outcome abstinence measure y, a binary intervention measure z, additional covariates $\boldsymbol{x} = (x_1, \dots x_p)$, and a binary indicator for response r where $r = 1$ indicates we observe the full set of measures, including in particular the abstinence measure, whereas $r = 0$ denotes we do not see the value for the abstinence measure y. In general terms, we want to make inference about the conditional distribution for y given z and $\boldsymbol{x}$, denoted by $f(y \mid z, \boldsymbol{x})$. We assume we have a sample of n independent observations, with sample values $(y_i, z_i, \boldsymbol{x}_i, r_i)$ drawn from the joint distribution for these measures.

When the outcome y is fully observed we can estimate the form of the conditional distribution $f(y \mid z, \boldsymbol{x})$, under an assumed parametric family, by maximizing the log likelihood

$$L_c(\beta; y, z, \boldsymbol{x}) = \sum_1^n \log f(y_i \mid z_i, \boldsymbol{x_i};\ \beta).$$

For example, we could posit a logistic model for the conditional distribution of y of the form

$$logit\ (P(y_i = 1 \mid z_i, \boldsymbol{x_i};\ \beta)) = log \frac{P(y_i=1 \mid z_i, \boldsymbol{x_i};\ \beta)}{1-P(y_i=1 \mid z_i, \boldsymbol{x_i};\ \beta)} = \beta_0 + \beta_z z_i + \sum_j \beta_j x_{ij}\ .$$

For the samples considered here though we only have direct information about the conditional distribution given z, $\boldsymbol{x}$, and $r = 1$, denoted $f(y \mid z, \boldsymbol{x}, r = 1)$. We can express the joint distribution of the outcome and response status, conditional on z and $\boldsymbol{x}$ as $f(y, r \mid z, \boldsymbol{x}) = f(y \mid z, \boldsymbol{x})\, f(\, r \mid y, z, \boldsymbol{x})$. The first factor on the right hand side of this factorization is the conditional distribution of interest and the second is the ***selection model*** distribution for r, the conditional distribution for how response status is related to the outcome, intervention assignment, and the covariates. Since we do not have any observations on y when $r = 0$, and hence no direct information on $f(r_i = 0 | y_i, z_i, \boldsymbol{x_i};\ \alpha)$, rather than maximize the likelihood

$$L(\beta, \alpha; y, r, z, \boldsymbol{x}) = \sum_1^n \log(f(y_i \mid z_i, \boldsymbol{x_i};\ \beta) f(r_i | y_i, z_i, \boldsymbol{x_i};\ \alpha))$$

we can maximize the expected likelihood

$$E\big(L(\beta, \alpha; y, r, z, \boldsymbol{x})\big)$$
$$= \sum_{r_i=1} \log(f(y_i|\, z_i, \boldsymbol{x_i};\ \beta) f(r_i|y_i, z_i, \boldsymbol{x_i};\ \alpha))$$
$$+ \sum_{r_i=0} \sum_{y_i=j} (f(y_i|\, z_i, \boldsymbol{x_i};\ \beta) f(r_i|y_i, z_i, \boldsymbol{x_i};\ \alpha))\, f(y_i|r_i, z_i, \boldsymbol{x}_i)$$

where $f(r_i|y_i, z_i, \boldsymbol{x_i};\ \alpha) = \frac{f(y_i|z_i, x_i; \beta) f(r_i|y_i, z_i, x_i;\, \alpha)}{\sum_{y=j} f(y\,|z_i, x_i;\, \beta) f(r_i|y, z_i, x_i;\, \alpha)}$. For a posited form for the selection model

$$logit\, f(\, r_i = 1 \mid y_i, z_i, \boldsymbol{x}_i) = \ logit\, P(r_i = 1\, |y_i, z_i, \boldsymbol{x}_i) = g(y_i, z_i, \boldsymbol{x}_i;\ \alpha)$$

for some g and α, together with the posited model for abstinence of the form

$$logit\ (P(y_i = 1 \mid z_i, \boldsymbol{x_i};\ \beta)) = \beta_0 + \ \beta_z\, z_i + \ \sum_j \beta_j x_{ij}$$

we can then find the maximum expected likelihood estimates for the joint model parameters using the EM algortihm of Ibrahim and Lipsitz {Ibrahim J.G. and Lipsitz S.R. *Parameter estimation from incomplete data in binomial regression when the missing data mechanism is nonignorable.* Biometrics 1996: 52(3); 1071-1078}. However, our inferential focus would be placed on the parameters in this latter conditional distribution for y given z and $\boldsymbol{x}$.

Selection Model Analyses. For the analysis of the primary outcome of prolonged abstinence, we fit a stratified logistic regression modeling the odds that a participant reported 6-months prolonged abstinence at one-year follow-up using intervention group, age, gender, and MHCP program strata as explanatory variables. To assess the potential impact of an informative nonresponse bias on the results of this initial analysis we fit a series of these selection model analyses wherein we posited different assumptions for how follow-up survey response would be related to the abstinence outcome, intervention assignment, and the sampling strata but, generally, the same model relating the outcome to intervention assignment and the sampling strata.

Selection Analysis Models 1, 2. The first two selection model analyses fit a simple logistic regression model for abstinence of the form

$$logit\ (P(y_i = 1 \mid z_i, s_i, \boldsymbol{x_i};\ \beta)) = \ \beta_0 + \ \beta_z\, z_i + \beta_{s_i} s_i + \ \sum_j \beta_j x_{ij}$$

where in addition to prolonged abstinence y, intervention z, potential covariates $\boldsymbol{x}$, we add the twelve level sampling strata, s. These twelve strata comprise the twelve combinations of sex, medical care program, and the three level age strata used in sampling individuals from the target population. The first selection model analysis fit the selection model

$$logit\ (P(r_i = 1 \mid\ y_i, z_i, s_i, \boldsymbol{x_i};\ \alpha)) = \ \alpha_0 + \ \alpha_y y_i + \alpha_z z_i + \alpha_{s_i} s_i + \ \sum_j \alpha_j x_{ij}$$

while the second analysis added an interaction between abstinence and intervention

$$logit\ (P(r_i = 1 \mid \ y_i, z_i, s_i, \boldsymbol{x_i};\ \alpha)) = \ \alpha_0 + \ \alpha_y y_i + \alpha_z z_i + \alpha_{yz} y_i z_i + \alpha_{s_i} s_i + \ \sum_j \alpha_j x_{ij}$$

Selection Analysis Models 3 and 4. We then modified these two analyses replacing the joint stratum measure with the individual age (a), sex (g), and healthcare coverage program (p) measures in an additive fashion. We fit a simple logistic regression model for abstinence of the form

$$logit\ (P(y_i = 1 \mid z_i, a_i, g_i, p_i, \boldsymbol{x_i};\ \beta)) = \ \beta_0 + \beta_z z_i + \beta_a a_i + \ \beta_s g_i + \ \beta_p p_i + \sum_j \beta_j x_{ij}.$$

The third selection model analysis fit the selection model

$$logit\ (\ P(r_i = 1 \mid y_i, z_i, a_i, g_i, p_i, \boldsymbol{x_i};\ \alpha)) \\ = \ \alpha_0 + \ \alpha_y y_i + \alpha_z z_i + \alpha_a a_i + \ \alpha_s g_i + \ \alpha_p p_i + \sum_j \alpha_j x_{ij}$$

while the fourth analysis again added an interaction between abstinence and intervention

$$logit\ (\ P(r_i = 1 \mid y_i, z_i, a_i, g_i, p_i, \boldsymbol{x_i};\ \alpha)) \\ = \ \alpha_0 + \ \alpha_y y_i + \alpha_z z_i + \alpha_{yz} y_i z_i + \alpha_a a_i + \ \alpha_s g_i + \ \alpha_p p_i + \sum_j \alpha_j x_{ij}$$

Selection Analysis Models 5 through 11. We then further modified this fourth selection model analysis to add different combinations of second order interactions between abstinence (y) and these three sampling strata measures.

In addition to the age, gender, and healthcare coverage program strata the models included the following measures from the baseline survey; participant race, education level, any children in home, cigarettes smoked per day, type of cigarettes smoked, time to first cigarette upon waking, age started smoking, maximum cigarettes per day over duration of smoking, any quit attempt over the past year, longest period without smoking, any prior NRT use, contemplation ladder score, self-reported confidence in quitting, number of friends and family that smoke, presence of home smoking rules, self-reported general health, days of alcohol use over prior month, difficulty living on household income, and two measures assessing how often over the prior two weeks the participant was bothered by i) having little pleasure in doing things and ii) feeling down. This collection of measures includes those that differed between respondents and non-respondents to the follow-up survey as well as key predictors of smoking status.

For each of these selection models we implemented the EM algorithm of Ibrahim and Lipsitz to estimate the parameters $\hat{\beta}$ and $\hat{\alpha}$ that maximized the expected likelihood and used the corresponding methods of Ibrahim and Lipsitz for estimating the standard errors for these estimates and for implementing a Wald test for the effect of the intervention on the abstinence measure. For each selection model scenario we constructed the AIC statistics for the resulting models for the abstinence measure and for the response (survey response) model in order to compare the fit of the models and identify the more plausible models. The models with lower AIC for the abstinence model tended to have more plausible estimated abstinence rates than the models that had lower AIC for the response model but higher AIC for the abstinence model. We

therefore deemed the models with the lower AIC for the abstinence model to be the more informative, more plausible, models.

Results for Prolonged Abstinence.

Table 1 summarizes the results for the 11 selection model analyses fit for the prolonged abstinence outcome. Specifically the table presents the AIC statistics for the fitted response and abstinence models, the abstinence model estimated parameter for the intervention and its standard error, corresponding odds ratio for the intervention and corresponding 95% confidence interval, p-value for the Wald test of the significance of the estimated intervention effect, and LS mean type model estimates for the abstinence rates for the control group and the intervention group. These estimates are not weighted to reflect the overall population from which the sample was drawn as in the main analysis but are simply LS mean type estimates for the sample, derived using the derived weights for the potential outcomes for those with missing data, to gauge estimated rates for a typical member of the sample as a proxy for these population estimates.

We highlight the models with the lower AIC statistics for the abstinence model with dark shading; for these three models the results are consistent with the analysis of the observed data, the odds ratios are increased relative to the main analysis which stems from the reduction in the estimated rates of abstinence in both arms though the estimated absolute differences in abstinence rates for a typical member of the sample are on par with the estimated difference in rates from the main analysis.

Results for 30 Day Abstinence.

Table 2 summarizes the results for the 11 selection model analyses fit for the 30 day abstinence outcome. Again, the table presents the AIC statistics for the fitted response and abstinence models, the abstinence model estimated parameter for the intervention and its standard error, corresponding odds ratio for the intervention and corresponding 95% confidence interval, p-value for the Wald test of the significance of the estimated intervention effect, and LS mean type model estimates for the abstinence rates for the control group and the intervention group. We again highlight the models with the lower AIC statistics for the abstinence model with dark gray shading. The models with low AIC are generally consistent with main results with estimated odds ratios ranging from 1.12 to 1.34 with p-values for the significance of the intervention effect ranging from 0.030 to 0.383. However the two models with the lowest AIC indicate that the intervention is likely effective in increasing 30 day abstinence with estimated odds ratios or 1.33 (1.02–1.74) and 1.34 (1.03–1.75) with p-values of 0.033/0.030.

Results for 7 Day Abstinence.

Table 3 summarizes the results for the 11 selection model analyses fit for the 7 day abstinence outcome. Again, the table presents the AIC statistics for the fitted response and abstinence models, the abstinence model estimated parameter for the intervention and its standard error, corresponding odds ratio for the intervention and corresponding 95% confidence interval, p-value for the Wald test of the significance of the estimated intervention effect, and LS mean type model estimates for the abstinence rates for the control group and the intervention group. We

highlight the models with the lower AIC statistics for the abstinence model with dark gray shading. These three models are consistent in indicating little evidence of an intervention effect on 7 day abstinence.

Table 1. Selection Model Results for Prolonged Abstinence

	R Model AIC	Y Model AIC	Est β	SE β	OR Est	OR CI Lower Bd	OR CI Upper Bd	p-value	$\hat{p}_C$	$\hat{p}_T$
**Extended Model 1**<br>Logit(responder) = a_0 + a_p strata + a_1 intervention + a_2 abstinence	1848	2806	0.571	0.097	1.770	1.463	2.141	<.0001	0.227	0.342
**Extended Model 2**<br>Logit(responder) = a_0 + a_p strata + a_1 intervention + a_2 abstinence + a_3 intervention abstinence	1800	2827	0.620	0.098	1.860	1.536	2.251	<.0001	0.228	0.354
**Extended Model 3**<br>Logit(responder) = a_0 + a_a age + a_m male + a_p program + a_1 intervention + a_2 abstinence	1822	2821	0.553	0.096	1.739	1.439	2.100	<.0001	0.233	0.346
**Extended Model 4**<br>Logit(responder) = a_0 + a_a age + a_m male + a_p program + a_2 abstinence + a_1 intervention + a_3 abstinence intervention	1805	2830	0.565	0.097	1.759	1.454	2.128	<.0001	0.234	0.350
**Extended Model 5**<br>Logit(responder) = a_0 + a_a age + a_m male + a_p program + a_2 abstinence + a_1 intervention + a_3 abstinence intervention + a_{3a} abstinence age + a_{3m} abstinence male + a_{3p} abstinence program	1244	3006	0.660	0.092	1.935	1.616	2.317	<.0001	0.269	0.416
**Extended Model 6**<br>Logit(responder) = a_0 + a_a age + a_m male + a_p program + a_2 abstinence + a_1 intervention + a_3 abstinence intervention + a_{3a} abstinence age	2640	1736	0.422	0.134	1.525	1.174	1.981	0.002	0.078	0.114
**Extended Model 7**<br>Logit(responder) = a_0 + a_a age + a_m male + a_p program + a_2 abstinence + a_1 intervention + a_{3m} abstinence male + a_3 abstinence intervention	1473	2941	0.654	0.094	1.922	1.599	2.311	<.0001	0.254	0.396
**Extended Model 8**<br>Logit(responder) = a_0 + a_a age + a_m male + a_p program + a_2 abstinence + a_1 intervention + a_3 abstinence intervention + a_{3p} abstinence program	2684	1890	0.520	0.128	1.682	1.309	2.160	0.000	0.090	0.142
**Extended Model 9**<br>Logit(responder) = a_0 + a_a age + a_m male + a_p program + a_2 abstinence + a_1 intervention + a_3 abstinence intervention + a_{3m} abstinence male + a_{3p} abstinence program	1244	3007	0.657	0.092	1.930	1.611	2.311	<.0001	0.269	0.416
**Extended Model 10**<br>Logit(responder) = a_0 + a_a age + a_m male + a_p program + a_2 abstinence + a_1 intervention + a_3 abstinence intervention + a_{3a} abstinence age + a_{3m} abstinence male	1538	2920	0.649	0.094	1.915	1.591	2.304	<.0001	0.249	0.389
**Extended Model 11**<br>Logit(responder) = a_0 + a_a age + a_m male + a_p program + a_2 abstinence + a_1 intervention + a_3 abstinence intervention + a_{3a} abstinence age + a_{3p} abstinence program	2645	1729	0.406	0.134	1.501	1.154	1.951	0.002	0.078	0.112

$\hat{p}_C$ is the LS mean type estimated abstinence rate in the control group, $\hat{p}_T$ is the LS mean type estimated abstinence rate in the intervention group

Table 2. Selection Model Results for 30 Day Abstinence

	R Model AIC	Y Model AIC	Est β	SE β	OR Est	OR CI Lower Bd	OR CI Upper Bd	p-value	$\hat{p}_C$	$\hat{p}_T$
**Extended Model 1**<br>Logit(responder) = $a_0 + a_p$ strata + a_1 intervention + a_2 abstinence	2116	2651	0.473	0.101	1.605	1.316	1.958	<.0001	0.201	0.287
**Extended Model 2**<br>Logit(responder) = $a_0 + a_p$ strata + a_1 intervention + a_2 abstinence + a_3 intervention abstinence	2098	2662	0.516	0.102	1.675	1.371	2.047	<.0001	0.199	0.294
**Extended Model 3**<br>Logit(responder) = $a_0 + a_a$ age + a_m male + a_p program + a_1 intervention + a_2 abstinence	2692	1763	0.230	0.133	1.259	0.971	1.633	0.083	0.090	0.111
**Extended Model 4**<br>Logit(responder) = $a_0 + a_a$ age + a_m male + a_p program + a_2 abstinence + a_1 intervention + a_3 abstinence intervention	2693	1762	0.152	0.133	1.164	0.897	1.510	0.254	0.093	0.107
**Extended Model 5**<br>Logit(responder) = $a_0 + a_a$ age + a_m male + a_p program + a_2 abstinence + a_1 intervention + a_3 abstinence intervention + a_{3a} abstinence age + a_{3m} abstinence male + a_{3p} abstinence program	2512	1681	0.287	0.135	1.333	1.024	1.736	0.033	0.078	0.101
**Extended Model 6**<br>Logit(responder) = $a_0 + a_a$ age + a_m male + a_p program + a_2 abstinence + a_1 intervention + a_3 abstinence intervention + a_{3a} abstinence age	2435	2373	0.281	0.110	1.324	1.068	1.643	0.011	0.167	0.210
**Extended Model 7**<br>Logit(responder) = $a_0 + a_a$ age + a_m male + a_p program + a_2 abstinence + a_1 intervention + a_{3m} abstinence male + a_3 abstinence intervention	2683	1726	0.117	0.134	1.124	0.864	1.463	0.383	0.091	0.101
**Extended Model 8**<br>Logit(responder) = $a_0 + a_a$ age + a_m male + a_p program + a_2 abstinence + a_1 intervention + a_3 abstinence intervention + a_{3p} abstinence program	2560	2110	0.487	0.119	1.627	1.289	2.055	<.0001	0.117	0.177
**Extended Model 9**<br>Logit(responder) = $a_0 + a_a$ age + a_m male + a_p program + a_2 abstinence + a_1 intervention + a_3 abstinence intervention + a_{3m} abstinence male + a_{3p} abstinence program	2581	2056	0.532	0.121	1.702	1.342	2.158	<.0001	0.108	0.170
**Extended Model 10**<br>Logit(responder) = $a_0 + a_a$ age + a_m male + a_p program + a_2 abstinence + a_1 intervention + a_3 abstinence intervention + a_{3a} abstinence age + a_{3m} abstinence male	2367	2450	0.308	0.108	1.360	1.102	1.680	0.004	0.178	0.227
**Extended Model 11**<br>Logit(responder) = $a_0 + a_a$ age + a_m male + a_p program + a_2 abstinence + a_1 intervention + a_3 abstinence intervention + a_{3a} abstinence age + a_{3p} abstinence program	2515	1677	0.294	0.135	1.342	1.030	1.748	0.030	0.077	0.101

$\hat{p}_C$ is the LS mean type estimated abstinence rate in the control group, $\hat{p}_T$ is the LS mean type estimated abstinence rate in the intervention group

Table 3. Selection Model Results for 7 Day Abstinence

	R Model AIC	Y Model AIC	Est β	SE β	OR Est	OR CI Lower Bd	OR CI Upper Bd	p-value	$\hat{p}_C$	$\hat{p}_T$
**Extended Model 1** Logit(responder) = a_0 + a_p strata + a_1 intervention + a_2 abstinence	1909	2917	0.357	0.094	1.429	1.188	1.719	0.000	0.273	0.349
**Extended Model 2** Logit(responder) = a_0 + a_p strata + a_1 intervention + a_2 abstinence + a_3 intervention abstinence	1899	2918	0.425	0.095	1.530	1.269	1.844	<.0001	0.267	0.358
**Extended Model 3** Logit(responder) = a_0 + a_a age + a_m male + a_p program + a_1 intervention + a_2 abstinence	2665	1869	-0.011	0.127	0.989	0.772	1.267	0.929	0.113	0.112
**Extended Model 4** Logit(responder) = a_0 + a_a age + a_m male + a_p program + a_2 abstinence + a_1 intervention + a_3 abstinence intervention	2667	1870	-0.011	0.127	0.989	0.772	1.268	0.932	0.113	0.112
**Extended Model 5** Logit(responder) = a_0 + a_a age + a_m male + a_p program + a_2 abstinence + a_1 intervention + a_3 abstinence intervention + a_{3a} abstinence age + a_{3m} abstinence male + a_{3p} abstinence program	1644	2978	0.518	0.093	1.678	1.398	2.014	<.0001	0.277	0.391
**Extended Model 6** Logit(responder) = a_0 + a_a age + a_m male + a_p program + a_2 abstinence + a_1 intervention + a_3 abstinence intervention + a_{3a} abstinence age	2200	2762	0.252	0.099	1.286	1.059	1.562	0.011	0.245	0.294
**Extended Model 7** Logit(responder) = a_0 + a_a age + a_m male + a_p program + a_2 abstinence + a_1 intervention + a_{3m} abstinence male + a_3 abstinence intervention	2628	1880	-0.033	0.126	0.968	0.756	1.238	0.793	0.116	0.113
**Extended Model 8** Logit(responder) = a_0 + a_a age + a_m male + a_p program + a_2 abstinence + a_1 intervention + a_3 abstinence intervention + a_{3p} abstinence program	1835	2902	0.478	0.095	1.613	1.338	1.943	<.0001	0.260	0.361
**Extended Model 9** Logit(responder) = a_0 + a_a age + a_m male + a_p program + a_2 abstinence + a_1 intervention + a_3 abstinence intervention + a_{3m} abstinence male + a_{3p} abstinence program	1621	2985	0.531	0.093	1.700	1.417	2.040	<.0001	0.278	0.395
**Extended Model 10** Logit(responder) = a_0 + a_a age + a_m male + a_p program + a_2 abstinence + a_1 intervention + a_3 abstinence intervention + a_{3a} abstinence age + a_{3m} abstinence male	2639	1874	0.045	0.126	1.046	0.817	1.340	0.719	0.111	0.115
**Extended Model 11** Logit(responder) = a_0 + a_a age + a_m male + a_p program + a_2 abstinence + a_1 intervention + a_3 abstinence intervention + a_{3a} abstinence age + a_{3p} abstinence program	1837	2905	0.460	0.095	1.584	1.314	1.908	<.0001	0.261	0.359

$\hat{p}_C$ is the LS mean type estimated abstinence rate in the control group, $\hat{p}_T$ is the LS mean type estimated abstinence rate in the intervention group
